# Case Report: Kounis syndrome associated with urticaria following COVID-19 infection

**DOI:** 10.3389/fcvm.2025.1542223

**Published:** 2025-04-04

**Authors:** Xia Li, Ailin Cao, Chengji Wang, Qun Guo, Xueying Chen, Yinghua Cui, Ying Gu

**Affiliations:** Department of Cardiology, Affiliated Hospital of Jining Medical University, Jining, China

**Keywords:** urticaria, allergy, Kounis syndrome, COVID-19, acute myocardial infarction

## Abstract

This case report describes a 58-year-old woman who sought treatment in the dermatology department after experiencing a three-day episode of widespread rash and itching, along with fever, chills, abdominal distress, and increased urinary frequency and urgency. Upon examination, she exhibited numerous erythematous patches and wheals on her face and body, devoid of blisters or erosions. Laboratory tests indicated an elevated white blood cell count, C-reactive protein, and serum amyloid A, while liver and kidney function tests were within normal limits. An electrocardiogram demonstrated sinus rhythm with T-wave alterations and a V2R/S ratio greater than 1. Subsequent nucleic acid testing confirmed the presence of COVID-19 infection, prompting the initiation of anti-allergic and supportive therapies. Despite this, the patient went on to develop chest pain, which was accompanied by electrocardiographic signs of acute extensive anterior wall myocardial infarction and elevated troponin I levels. Coronary angiography subsequently revealed mild coronary artery stenosis, with no significant blockages or stenoses in the coronary arteries, leading to a diagnosis of Kounis syndrome type II. This case underscores the significance of considering Kounis syndrome in patients with a history of infection or allergies who present with chest pain, emphasizing the necessity for thorough clinical evaluation and continued research.

## 1 Introduction

Kounis Syndrome, characterized by the development of acute coronary syndrome following an allergic reaction, was first described by Kounis and colleagues in 1991 (1). The pathophysiology of this syndrome involves the activation of mast cells and platelets, rendering it a distinct and uncommon critical condition in clinical practice. While COVID-19 is primarily known for respiratory symptoms, it can also affect multiple organ systems, including the skin and cardiovascular system. Urticaria has been identified as a notable extra-pulmonary cutaneous manifestation of COVID-19 (2). Although there are reports of Kounis Syndrome triggered by urticaria (3, 4), there is limited literature on cases associated with COVID-19-related urticaria. This case report describes a 58-year-old female patient who developed urticaria subsequent to a COVID-19 infectionand later experienced recurrent coronary artery spasms, which were suspected to have contributed to an acute myocardial infarction. The patient's condition was managed successfully with a regimen including anti-allergic, anti-vasospastic, and antiviral treatments. This case emphasizes the importance of considering cardiovascular complications in the differential diagnosis of patients with COVID-19 who present with cardiac symptoms.

## 2 Case description

**Figure 1:**
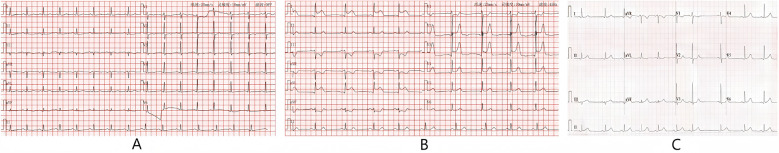
**(A)** ECG of the patient on admission: T-wave changes; **(B)** ECG at the onset of chest pain: ST segment elevation in leads I, aVL, V2–5; **(C)** after the patient chest pain was relieved, the elevated ST segment fell back and premature atrial contractions occurred.

**Figure 2:**
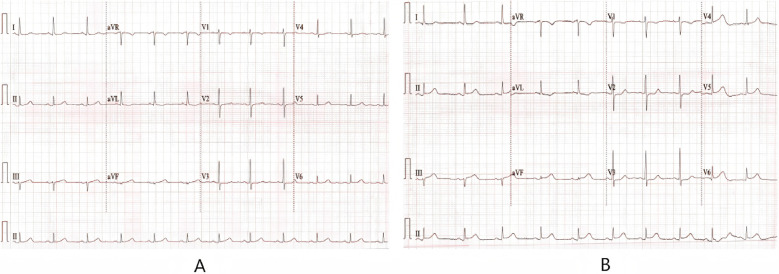
**(A)** When the patient has another angina attack. **(B)** Electrocardiogram when the pain patient has relief from chest.

**Figure 3:**
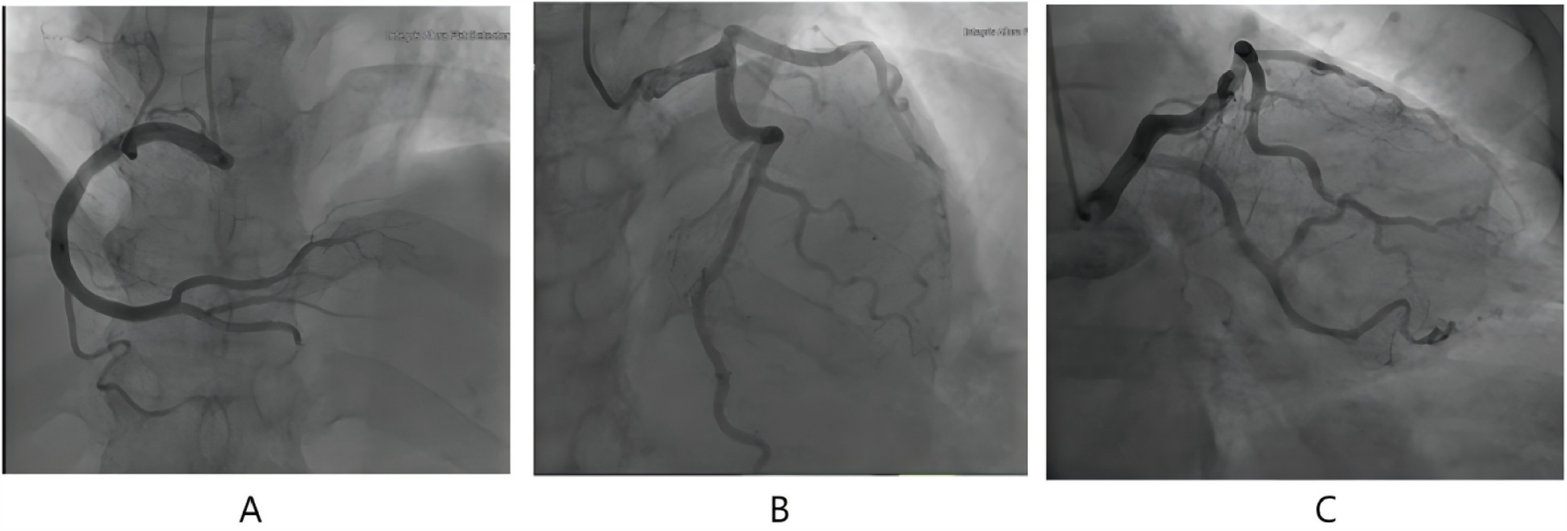
Angiographic image of this patient. **(A)** (CRA25.5+RAO1.7) the right coronary artery. **(B)** (CRA39.2+RAO3.2) the left anterior descending branch. **(C)** (CAU23.1+RAO23.6) the circumflex branch.

The patient, female, 58 years old, was admitted to the dermatology department of our hospital on August 1, 2024 due to “generalized rash accompanied by itching for 3 days and frequent urination and urgency for 2 days”. Three days before admission, the patient developed generalized erythema and wheals without obvious inducement, accompanied by itching, fever, chills, abdominal discomfort. The highest recorded body temperature was 39.3 °C. There was a little nausea but no vomiting or chest tightness. She was tested negative for COVID-19 nucleic acid at Nanjing Drum Tower Hospital. Blood routine and CRP tests were normal. She was treated with ReDuNing Injection (a traditional Chinese medicine preparation) and Loratadine Tablets. The body temperature returned to normal but the rash did not subside. Two days later, the patient developed frequent urination, urgency and pain without hematuria. She was treated with “Dexamethasone Sodium Phosphate Injection” and “Levofloxacin”. The rash still did not improve. She had a 20-year history of “hypertension” and usually took “Amlodipine Besylate Tablets” to control blood pressure normally. In 2014 and 2016, the patient underwent coronary CTA examination on two occasions, which revealed no significant obstruction or stenosis in the coronary arteries. She denied a history of drug or food allergy and family genetic disease. She had a history of contact with COVID-19. Physical examination: Multiple erythema and wheals on the face and body, fused into patches, without blisters or erosion. Physical examination of heart and lungs was normal. Blood routine, CRP and serum amyloid A were abnormal (Table 1); immunoglobulin E was 532 IU/ml; urine routine white blood cell count was 24.42/ul; liver and kidney function and myocardial injury markers were normal; mycoplasma pneumoniae antibody and allergens were negative. The electrocardiogram on admission showed (1) sinus rhythm, (2) T wave changes, and (3) V2R/S greater than 1 (Figure 1A); echocardiography showed a left ventricular ejection fraction (LVEF) of 63%; mild tricuspid regurgitation and decreased left ventricular diastolic dysfunction. After admission, she was given Methylprednisolone Sodium Succinate for Injection (40 mg, twice a day), Loratadine (10 mg, once a day), and Ebastine Tablets (10 mg, once a day) for anti-allergy, Omeprazole for Injection (40 mg, twice a day) for protecting gastric mucosa, Levofloxacin and Sodium Chloride Injection (0.5 g, once a day) for anti-infection, and Amlodipine Besylate Tablets (5 mg, once a day) for lowering blood pressure and other symptomatic and supportive treatments. The generalized rash and itching were alleviated. Five days later, the dose of Methylprednisolone Sodium Succinate for Injection was reduced (20 mg, twice a day). Six days later, the patient developed cough and chest tightness. The body temperature was normal. Seven days later, the nucleic acid test for six respiratory pathogens was all negative. The nucleic acid test for novel coronavirus was positive again. The patient was treated with simnotrelvir 0.75 g + ritonavir 0. 1 g (twice a day for 5 days). Nine days later, the patient felt compressive pain in the precordial area. The NRS pain score was 7 points, accompanied by profuse sweating that did not relieve continuously. Electrocardiogram indicated (1) sinus bradycardia; (2) acute extensive anterior wall myocardial infarction; (3) ST-T changes (Figure 1B); Myocardial injury marker troponin I was 0.072 ng/ml. The patient previously underwent two coronary CT angiographies, both of which showed no significant stenosis. This episode of chest pain occurred at rest during nighttime, leading us to suspect acute myocardial infarction associated with coronary artery spasm. Considering the patient's history of allergic reactions and the potential risk of contrast agents used in coronary angiography exacerbating allergic responses, coronary angiography was not performed. Instead, the patient was treated as follows: Aspirin enteric-coated tablets (100 mg, once daily) and clopidogrel bisulfate tablets (75 mg, once daily) were administered for dual antiplatelet therapy. Heparin (0.4 ml, twice daily) was used for anticoagulation. To relieve coronary artery spasm, diltiazem hydrochloride tablets (30 mg four times daily) and nicorandil tablets (5 mg three times daily) were prescribed. Additionally, intravenous methylprednisolone sodium succinate (10 mg once daily) was administered to mitigate potential allergic reactions, and isosorbide dinitrate injection (20 mg once daily) was given to further alleviate coronary spasm and improve myocardial perfusion. Following this medical treatment, the chest pain symptoms were relieved, and the electrocardiogram returnedto normal. However, atrial premature beats were observed (Figure 1C). Ten days later, reexamination of echocardiography showed no definite segmental wall motion abnormality in resting state, and LVEF was 60%. Considering the patient's allergic history, the risks associated with coronary angiography, and the symptomatic relief achieved with medication, a conservative drug treatment approach was continued. Methylprednisolone sodium succinate was gradually reduced and changed to prednisone acetate tablets (20 mg, once daily) orally to inhibit inflammatory reaction. Two weeks later, the patient had compressive pain in the chest and back again. The NRS pain score was 4 points, accompanied by radiating pain to the left shoulder and jaw. After isosorbide nitrate and papaverine hydrochloride were pumped in, the symptoms were not significantly relieved. Reexamination of electrocardiogram showed dynamic evolution of T waves in precordial leads (Figure 2), and myocardial injury marker troponin I was 0.093 ng/ml. Considering that the patient's chest pain symptoms did not relieve continuously, coronary angiography was performed on August 14, which indicated mild coronary artery stenosis (Figure 3). Continue to give antiplatelet aggregation, lipid regulation, anti-coronary artery spasm and anti-allergic treatment. Eighteen days later, reexamination of novel coronavirus nucleic acid test was negative. The rash all over the body gradually disappeared, and the patient was discharged after improvement. After discharge, the dosage of prednisone acetate tablets was gradually reduced and eventually discontinued. The patient continued to take nicorandil, diltiazem hydrochloride tablets, aspirin enteric-coated tablets and atorvastatin calcium tablets orally. At the one-month follow-up, no recurrence of urticaria or angina symptoms was observed.

**Table 1:** Changes of inflammatory indexes during the patient's onset process.

Laboratory test (Reference range)	Jul 31	Aug 2	Aug 3	Aug 6	Aug 8	Aug 10	Aug 14	Aug 18	Aug 21	Aug 23
CTnI (ng/ml) (0.01–0.023)	—	—	—	—	—	0.072	0.093	0.052	0.011	—
WBC × 10^9^/L (3.5–9.5)	11.55	12.3	14.5	16.19	22.04	—	—	13.73	9.75	8.23
CRP(mg/L) (0–6)	51.34	72.03	30.50	4.64	8.10	—	—	53.20	62.65	22.23
SAA(mg/L) (0–10.08)	474.29	528.00	531.40	59.41	64.24	—	—	—	—	—

cTnI, cardiac Troponin I; WBC, white blood cell count; CRP, C-reactive protein; SAA, serum amyloid.

## 3 Discussion

The main clinical symptoms of coronavirus disease 2019 (COVID-19) include fever, sore throat, fatigue, cough, and dyspnea. Some severe patients may experience respiratory failure, multiple organ dysfunction, and even death (5). With the increase in clinical cases, it is currently believed that COVID-19 can involve various systems throughout the body, including the skin and cardiovascular system. The etiology or inducement of urticaria is relatively complex. Its onset is related to infection. The specific pathogenesis may be the activation and degranulation of mast cells through immune and non-immune mechanisms (6). Literature reports that urticaria is an important part of the extrapulmonary skin manifestations of COVID-19 and can appear at different times during infection. It can precede, occur simultaneously with, or follow other symptoms. Watashi et al. reported a patient with acute urticaria as the first symptom of COVID-19 (7). There are also patients with fever and urticaria-like rashes as clinical manifestations (8). At the same time, the impact of COVID-19 on the cardiovascular system has attracted much attention. 50%–60% of patients with myocardial injury do not have severe coronary artery stenosis (9). Some scholars call this situation acute COVID-19 cardiovascular syndrome (10). COVID-19 can cause myocardial injury through various mechanisms, including direct viral infection of cardiomyocytes, excessive inflammatory responses, endothelial dysfunction, microthrombosis, plaque rupture, and hypoxemia leading to myocardial ischemia and cell apoptosis (11).

The incubation period of the new coronavirus is 1–14 days, mostly 3–7 days. Usually, nucleic acid can be detected positive 3–7 days after infection (12). This patient had rare urticaria with fever as the first clinical symptom. No special food or drugs had been taken before, so urticaria caused by food or drugs can be excluded. Although the first nucleic acid test for the new coronavirus was negative, considering that the new coronavirus may act as an allergen, induce an immune response in the body, activate mast cells to degranulate, trigger hypersensitivity reactions, release a large amount of inflammatory mediators, and cause urticaria. At the same time, inflammatory mediators will lead to endothelial dysfunction, microcirculatory dysfunction, and even damage the stability of atherosclerotic plaques to a certain extent, thereby inducing repeated coronary artery spasms. The latest literature reports (13) that Kounis syndrome is divided into four types: Type I occurs in patients with normal coronary angiography and is the most common type in clinical practice. Most cases are coronary artery spasms caused by allergic reactions. Type II is for those with coronary atherosclerosis. Inflammatory mediators induce coronary artery spasms, plaque rupture, and are often accompanied by thrombosis. Type III occurs in patients with in-stent thrombosis or restenosis of coronary arteries. Type IV occurs in patients who have previously undergone coronary artery bypass grafting. In this case, the patient was admitted to the hospital due to urticaria. The new coronavirus was positive. The immunoglobulin E level was increased. Subsequently, the patient had chest pain symptoms. The ST segment was transiently elevated on the electrocardiogram, and troponin was increased. Coronary angiography suggested the presence of coronary atherosclerosis. After giving anti-allergic and anti-spasm drugs, the symptoms were relieved. Combined with the medical history, clinical manifestations and auxiliary examinations, this patient is considered to have type II Kounis syndrome. Podder et al. (14) reported a rare case of ibuprofen-induced type 1 Kounis syndrome, with urticaria as the initial symptom, which is similar to our case where urticaria was also the primary clinical manifestation. However, the triggering factors differed between the two cases (COVID-19 infection vs. NSAID exposure). These cases highlight the importance of considering Kounis syndrome as a potential diagnosis when allergic patients present with cardiac symptoms.

Kounis syndrome needs to be differentiated from Takotsubo syndrome. The clinical manifestations and outcomes of these two diseases are similar. There are even reports that these two diseases can coexist, called ATAK syndrome (15). However, in Kounis syndrome, allergic reactions induce coronary artery spasms, plaque erosion, rupture or thrombosis. The allergic indicators in the patient's peripheral blood are often significantly increased. There may be no change in ventricular wall motion.

Takotsubo syndrome can be caused by various emotions and diseases. Current research believes that it is related to sympathetic nerve activation. The peripheral allergic indicators of patients usually do not change. Abnormal left ventricular wall motion is a necessary condition for the diagnosis of Takotsubo syndrome (16), and the local abnormal wall motion often exceeds the scope of the supply of a single epicardial vessel. In this patient, no obvious abnormal wall motion was seen on echocardiography, so Takotsubo syndrome is not considered.

In summary, allergic reactions triggered by various foods, medications, or environmental factors can induce Kounis syndrome. COVID-19, known to cause systemic inflammatory responses leading to multi-organ damage, may also contribute to this condition. When patients present with cardiac symptoms, it is crucial to recognize the importance of dynamic electrocardiogram monitoring and myocardial biomarker testing to ensure timely and accurate diagnosis, thereby reducing the risk of missed or misdiagnosed cases. Additionally, in future clinical practice, a comprehensive evaluation of potential triggering factors for Kounis syndrome is essential.

## 4 Conclusions

This case report describes a rare case of recurrent coronary artery spasm triggered by urticaria following COVID-19 infection, emphasizing the importance of considering Kounis syndrome in patients with a history of infection or allergies presenting with chest pain. This underscores the need for further research and vigilant clinical assessment.

## Data Availability

The raw data supporting the conclusions of this article will be made available by the authors, without undue reservation.
